# Positive selection acts on regulatory genetic variants in populations of European ancestry that affect ALDH2 gene expression

**DOI:** 10.1038/s41598-022-08588-0

**Published:** 2022-03-16

**Authors:** Helmut Schaschl, Tobias Göllner, David L. Morris

**Affiliations:** 1 Department of Evolutionary Anthropology, Faculty of Life Sciences, University of Vienna, Djerassiplatz 1, 1030 Vienna, Austria; 2 Department of Medical and Molecular Genetics, Faculty of Life Sciences and Medicine, King’s College London, Great Maze Pond, London, SE1 9RT UK

**Keywords:** Evolution, Genetics, Population genetics

## Abstract

ALDH2 is a key enzyme in alcohol metabolism that protects cells from acetaldehyde toxicity. Using iHS, iSAFE and *F*_*ST*_ statistics, we identified regulatory acting variants affecting *ALDH2* gene expression under positive selection in populations of European ancestry. Several SNPs (rs3184504, rs4766578, rs10774625, rs597808, rs653178, rs847892, rs2013002) that function as eQTLs for *ALDH2* in various tissues showed evidence of strong positive selection. Very large pairwise *F*_*ST*_ values indicated high genetic differentiation at these loci between populations of European ancestry and populations of other global ancestries. Estimating the timing of positive selection on the beneficial alleles suggests that these variants were recently adapted approximately 3000–3700 years ago. The derived beneficial alleles are in complete linkage disequilibrium with the derived *ALDH2* promoter variant rs886205, which is associated with higher transcriptional activity. The SNPs rs4766578 and rs847892 are located in binding sequences for the transcription factor *HNF4A,* which is an important regulatory element of *ALDH2* gene expression. In contrast to the missense variant *ALDH2* rs671 (ALDH2*2), which is common only in East Asian populations and is associated with greatly reduced enzyme activity and alcohol intolerance, the beneficial alleles of the regulatory variants identified in this study are associated with increased expression of *ALDH2*. This suggests adaptation of Europeans to higher alcohol consumption.

## Introduction

The Neolithic transition from a hunter-gatherer lifestyle to an agriculturist one, about 9000–13,000 years ago, included substantial changes in food processing and dietary habits associated with plant and animal domestication^1^. One of the key questions in biological anthropology is whether these changes resulted in selective pressure, influencing the expression of genes in the human genome. Identifying such loci has the potential to detect the underlying genetic variants contributing to the risk for various human diseases such as autoimmune diseases, cancer, or cardiovascular disease^1^. Alcohol consumption and culture-related drinking behavior is probably one of the major changes in human dietary habits and lifestyle over the last 10,000 years. Production of larger amounts of alcoholic beverages had probably begun by the early Neolithic. A recent study reports archaeological evidence for cereal-based beer brewing by the semi-nomadic Natufians at Raqefet Cave (Mount Carmel in the north of Israel) dating back 11,700–13,700 years ago^2^. Today, large amounts of alcohol are consumed in many societies. Recent data from the World Health Organization (WHO) show that worldwide about 3 million deaths and 132.6 million disability-adjusted life years are attributable to the harmful use of alcohol^3^. In particular, Europe stands out in the WHO data as the region with the highest alcohol consumption and the highest burden of alcohol-related diseases. Large amounts of episodic drinking (binge drinking) as well as chronic alcohol consumption are associated with several very harmful effects such as alcoholic liver disease, intestinal inflammation, cancer, hypertension, brain damage including adverse behavioural changes, and decreased fertility^4,5^. While heavy alcohol consumption can cause complex negative physiological effects, positive effects of light to moderate alcohol consumption have also been reported. Light to moderate alcohol consumption has been associated with a reduced risk of some forms of cardiovascular disease and autoimmune diseases^6–10^. The most commonly ingested alcohol is ethanol (EtOH, CH_3_CH_2_OH) which is absorbed from the gastrointestinal tract by passive diffusion. EtOH is oxidized in the first step, mainly in the liver, to acetaldehyde by alcohol dehydrogenases (ADH). The genes *ADH4*, *ADH1A*, *ADH1B* and *ADH1C*, which are located in an array in the region of chromosome 4q23, encode closely related proteins and carry out most of the EtOH oxidation in liver. Cytochrome P450 2E1 (*CYP2E1*) and the enzyme catalase (*CAT*) also participate in this metabolic pathway, albeit to a lesser extent. EtOH is also metabolized in non-liver tissue tissues such as the brain, mainly by the microsomal EtOH oxidation system (MEOS), involving the CYP2E1 enzyme^11^. In the second step of EtOH metabolism, acetaldehyde, which is a chemically reactive and toxic compound, is oxidized by aldehyde dehydrogenases (ALDHs) to acetate^12,13^.

Several studies provide evidence that recent positive selection acts on the *ADH1B* locus in Asian, European and African populations^14–19^. At this locus, two missense substitutions play a role at the SNPs rs1229984 (G > A; p.Arg48His) and rs2066702 (C > T; p.Arg370Cys). They express three different isoforms. The ADH1B*1 isoform with arginine at both codon positions is the most common allele globally, except in populations of East Asia ancestry. In East Asia, the derived allele ADH1B*2 (rs1229984) presents the common allele (with a frequency of about 0.70)^13^. The ADH1B*3 allele (rs2066702) occurs only in individuals of African ancestry, with allele frequencies ranging from 0.09 to 0.28^13^. The two derived isoforms ADH1B*2 and ADH1B*3 metabolise EtOH at about 11 and 3 times the rate of ADH1B*1, respectively^13^. Several studies report that rs1229984 in the *ADH1B* locus is associated with a reduced risk of alcoholism in different ancestries^20–27^. The positive selection on the derived allele was estimated to have occurred about 7000–15,000 years ago^16,28,29^, which overlaps with the time-frame of the origin and expansion of Neolithic agriculture in East Asia. Nonetheless, it remains unclear whether the driving selective force acting on this genetic polymorphism emanates from the protective effect against alcohol dependence or from the higher efficiency of this polymorphism in metabolizing EtOH.

The mitochondrial enzyme ALDH2 plays the key role in the second step of EtOH metabolism by converting acetaldehyde into acetate. ALDH2 is not only a major detoxification enzyme for EtOH-derived acetaldehyde, but is also involved in detoxifying reactive aldehydes derived from reactive oxygen species (ROS). Aldehydes are toxic molecules that can form genotoxic DNA- and protein-adducts in cells^30^. Accumulation of high levels of acetaldehyde can be mutagenic, carcinogenic^31–33^ and may negatively affects the immune system^34^. In contrast to the *ADH* genes, *ALDH2* is expressed in most human tissues, with high levels in the liver, heart, kidney, and muscle tissues^35^. In the coding region of *ALDH2*, the missense variant rs671 (G > A; p.Glu504Lys) expresses the isoforms ALDH2*1 and ALDH2*2. The ALDH2*2 variant is found only in individuals of East Asian ancestry, reaching frequencies of up to 40% in some East Asian populations such as Han Chinese and Japanese^13,36,37^. This allele significantly affects alcohol metabolism because it results in an inactive enzyme and thus an excess of the toxic acetaldehyde in cells, even with moderate alcohol consumption. The symptoms are severe facial flushing, nausea, headache and tachycardia^38^. East Asians homozygous for ALDH2*2 have a very low risk for alcohol dependency^26,38,39^. The ALDH2 enzyme plays a central role in protecting cells from EtOH toxicity by metabolizing acetaldehyde (and other endogenous aldehyde products), is anti-inflammatory^40^, and functions in myocardial protection^41–43^. Accordingly, this gene is of great biomedical interest. *ALDH2* is located at the human chromosomal region 12q24.12. Several genome-wide association studies (GWAS) have found this genomic region to be associated with multiple human diseases such as rheumatoid arthritis^44^, systemic lupus erythematosus^45^, type 1 diabetes^46^, hypertension^47^ and coronary artery disease^48^. This region, approximately 0.6 Mbp in size (according to the human reference genome), encompasses in addition to *ALDH2* the genes *CUX2, FAM109A, SH2B3, ATXN2, BRAP, ACAD10* and *MAPKAPK5*, as well as the uncharacterized transcript ENST00000546840.3 (UniProt F8VP50—Aldedh domain-containing protein), which partially overlaps with the genes *ACAD10* and *ALDH2*. High *F*_*ST*_ values at linked sites at the *ALDH2* locus point to some form of selection for this genomic region^36^. A recent study analysing rare singletons in the Japanese population identified the SNP rs3782886, which is in linkage disequilibrium (LD) with the missense SNP rs671 in the 12q24.12 region, as under recent positive selection^49^. In this study, we applied population genetic models of natural selection and included functional genetic data to identify the targets of positive selection in this genomic region. Several lines of evidence indicate that recent positive selection is acting on regulatory variants that influence *ALDH2* gene expression in populations of European ancestry.

## Results

### Positive selection in populations of European ancestry

**Figure 1:**
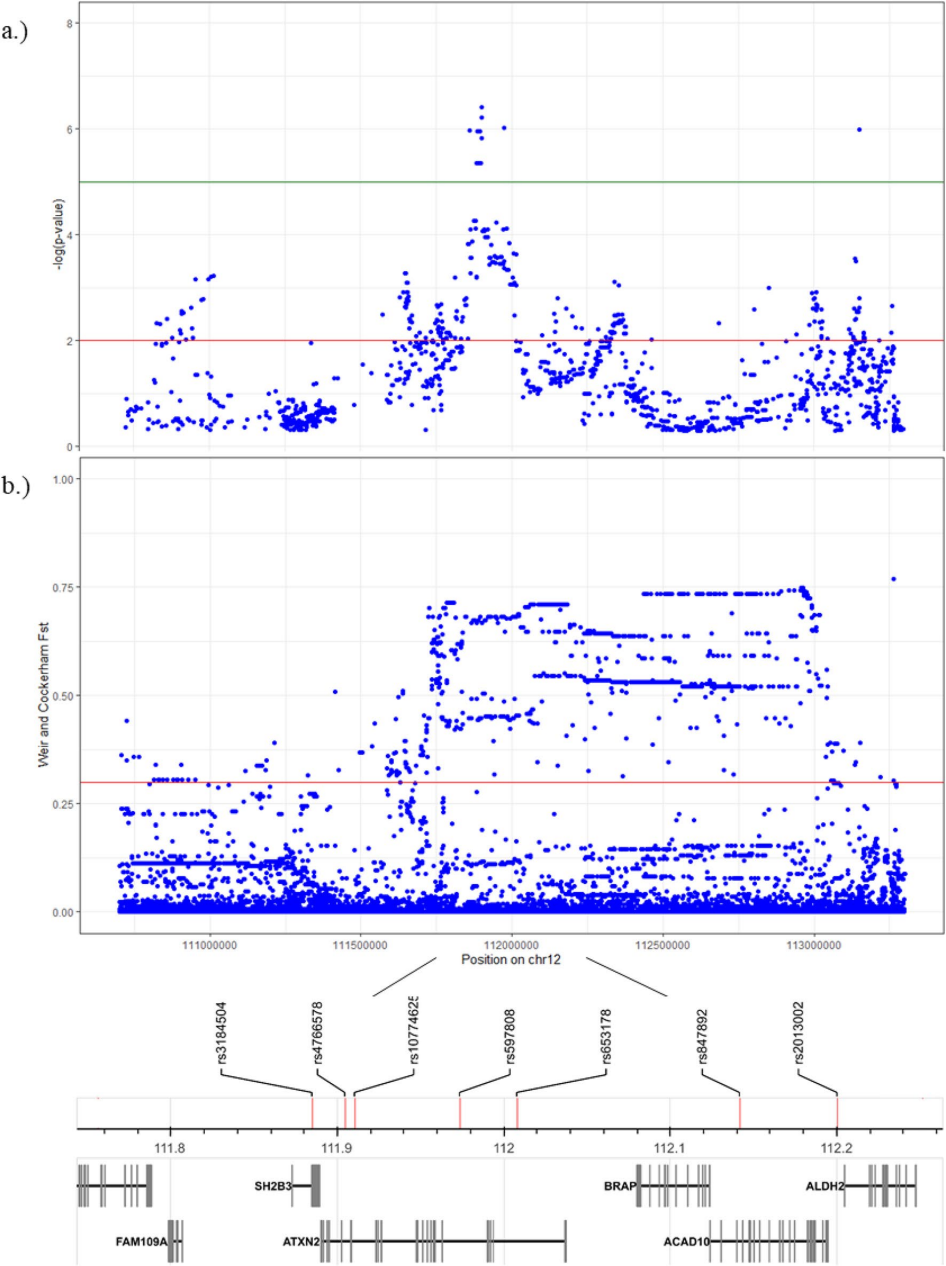
(**a**) iHS *p*-values plotted across the human chromosomal region 12.q24.12 for the population GBR (European genetic ancestry); red/green lines: threshold for significant (*p* < 0.01; Bonferroni correction *p* < 1 × 10^–5^) iHS scores; (**b**) pairwise *F*_*ST*_ (GBR–LWK); red line: significant outlier loci with *F*_*ST*_ > 0.3. Bottom: position of genes and SNPs from Table 1.

The iHS analysis shows evidence that the human chromosomal region 12.q24.12 is under positive selection in populations of European ancestry. Figure 1a plots the iHS scores in the European population GBR; Fig. 1b shows the pairwise *F*_*ST*_ values for GBR *vs*. the African population LWK across 12.q24.12. The red and green lines indicate significant (*p* < 0.01 and after Bonferroni correction *p* < 1 × 10^–5^, respectively) iHS scores and the genome-wide threshold (95% confidence level) for *F*_*ST*_ outlier loci (*F*_*ST*_ > 0.3).

### Positive selection acts on regulatory variants of ALDH2

**Figure 2:**
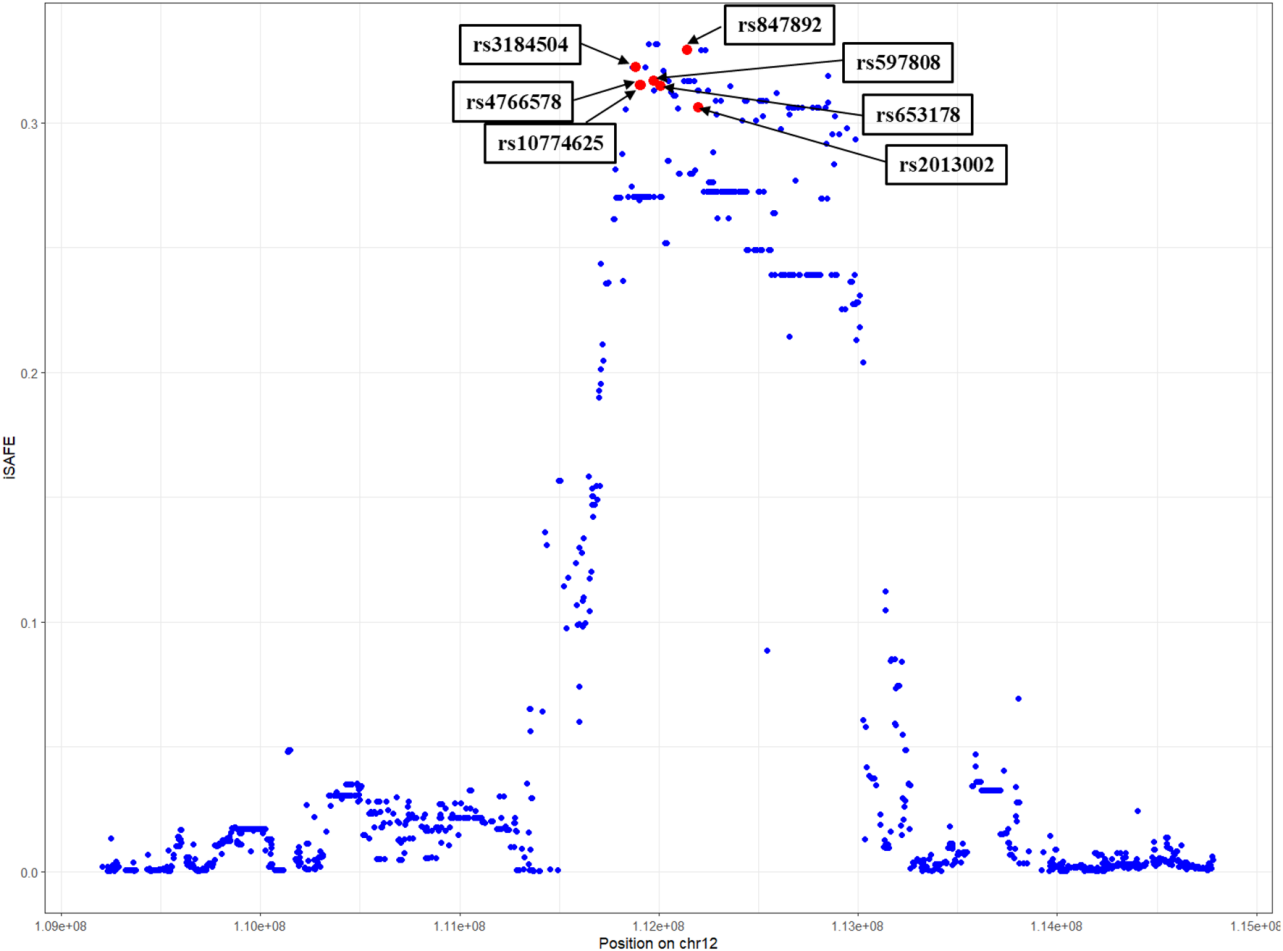
iSAFE scores plotted for SNPs surrounding the chr12q24.12 region (5.6 Mbp window) for the population GBR (European genetic ancestry); also indicated are the SNPs identified by the iHS statistics as being under positive selection; the top-ranked SNPs are above the threshold sores iSAFE > 0.304.

From the GTEx database, we obtained in total 1591 *cis*-QTLs that influence *ALDH2* gene expression (Supplementary Table 1); of these *cis*-QTLs, we identified 204, 217 and 53 eQTLs that had significant (*p* < 0.01) iHS scores in the European samples GBR, TSI and FIN, respectively (Supplementary Table 2). We also obtained *cis*-eQTLs (1970 in total) for the other protein-coding genes located in this genomic region (*CUX2, FAM109A, SH2B3, ATXN2, BRAP, ACAD10, MAPKAPK5*) (Supplementary Table 3). In contrast to the eQTLs for *ALDH2*, we did not obtain significant iHS values for these SNP eQTLs, except for SNPs that also function as eQTLs for *ALDH2*. We further identified seven SNPs (rs3184504, rs4766578, rs10774625, rs597808, rs653178, rs847892, rs2013002) that are under positive selection in European populations that have very large global locus-specific *F*_*ST*_ values > 0.3, i.e. are outlier loci (Table 1). The corresponding EHH plots and pairwise *F*_*ST*_ values of these SNPs can be found in Supplementary Fig. 1 and Supplementary Table 4, respectively. The pairwise *F*_*ST*_ values for these SNPs comparing populations of European ancestries *vs*. African, East Asian and South Asian ancestries ranged from 0.253 to 0.691. The iSAFE analysis also identified several SNPs in the chr12q24.12 region under positive selection (Supplementary Table 5). Moreover, the identified seven SNPs under positive selection (by the iHS and *F*_*ST*_ statistics) were also identified by iSAFE as top-ranked mutations with iSAFE scores > 0.304, i.e., above the significant threshold (Fig. 2). These SNPs function as eQTLs for *ALDH2,* and the beneficial alleles are associated with increased *ALDH2* gene expression in various human tissues (according to the set of tissues represented in GTEx) such as esophagus—mucosa, skin, muscle—skeletal, brain—nucleus accumbens, artery—tibial, artery—aorta, and thyroid. The average allele frequencies for these SNPs are given in Table 1; for most of these SNPs the frequency of the derived beneficial alleles reaches almost 50% in the European populations. In contrast, the derived alleles are very rare (< 0.3%) in African and East Asian ancestries and at low frequency in populations of South Asian ancestry (< 7% with the exception of rs847892). We also compared the allele frequency at these loci with ancient Eurasians, including ancient hunter-gatherers (8.2–7.5 kya) from the study of^50^. The allele frequency data from the latter study show for the SNPs rs3184504, rs4766578, rs10774625, and rs653178 that the ancestral alleles were fixed in ancient European hunter-gatherers. As expected, the Neanderthal and Denisovan data on the UCSC Genome Browser also show only ancestral alleles at these loci. In contrast, early European farmers (8.4–4.2 kya) and individuals with steppe ancestry (5.4–3.6 kya) had frequencies between 8 and 25% of the derived alleles at these loci. The analysis of the selection coefficient (*s*) revealed that *s* ranged from 0.04 to 0.1, suggesting very strong positive selection acting on these SNP eQTLs (Table 1). The corresponding allele trajectory plots, inferred by the Clues method, are presented in Supplementary Figure S2. In the European sample GBR we estimated the timing of positive selection (using the method Startmrca) of the derived beneficial alleles to be from about 3.0 to 3.7 kya with the exception of SNP rs847892, which we date at 6.0 kya (Table 1). This range of estimates are very similar to the TMRCA estimates calculated for the other European samples (TSI and FIN) (Supplementary Table 6). We also calculated the TMRCA for the derived allele of the East Asian-specific polymorphism (missense variants) at rs671-A/G and rs3782886-C/T in the East Asian population CHB, which yielded an estimation of 5.8 kya (CI: 4.8–6.7) and 5.4 kya (CI: 3.3–6.5), respectively. In addition, we used the method Clues to obtaining allele ages for the seven SNPs under positive selection. Clues calculated a similar timing of selection (2.6 kya to 4.5 kya) for the SNPs rs3184504, rs4766578 and rs10774625 as the Startmrca method (Supplementary Table S7). However, the timing of selection for the SNPs rs597808, rs653178 and rs2013002 was estimated to much older ages ranging in time frames from 7.4 kya to 14.1 kya; rs847892 between 21.3 to 30.1 kya.

**Table 1:** SNPs under positive selection at the human chromosomal region 12q24.12 in populations with European ancestry (GBR, TSI, FIN). Given are iHS scores and the calculated (-log) *p*-values (in bold Bonferroni correction with* p* < 1 × 10^–5^), the timing (*t*) of positive selection on the derived beneficial allele in thousand years ago (kya) and 95% credible interval (CI) (rounded to one decimal figure), the estimated selection coefficients (*s*) in GBR, average allele frequency in % for the derived beneficial allele/ancestral allele in the different ancestries and global locus-specific *F*_*ST*_ values (sd = standard deviation) calculated across all analysed populations.

Beneficial allele/ ancestral allele	Location	iHS			iHS -logp	*s* (logLR)	*t* (kya)	95% CI	Allele frequency in %				Locus-specific *F*_*ST*_ (sd)
		GBR	TSI	FIN					AFR	EUR	SAS	EAS	
rs3184504-T/C	Exon, *SH2B3*	− 3.2	− 3.3	− 2.9	3.2; 3.3; 2.7	0.1 (93.7)	3.7	3.2–4.3	0.2/99.8	46/54	7/93	0.2/99.8	0.351 (0.060)
rs4766578-T/A	Intron, *ATXN2*	− 3.8	− 3.1	− 3.1	4.1; 3.0; 3.0	0.1 (89.3)	3.5	3.0–4.0	0.2/99.8	48/52	7/93	0.2/99.8	0.366 (0.063)
rs10774625-A/G	Intron, *ATXN2*	− 3.8	− 3.1	− 3.0	4.1; 3.0; 2.9	0.1 (89.9)	3.0	2.7–3.4	0.2/99.8	48/52	7/93	0.2/99.8	0.366 (0.062)
rs597808-A/G	Intron, *ATXN2*	− 3.8	− 4.2	− 3.1	4.1; 4.8; 3.0	0.09 (69.9)	3.5	3.0–4.1	0.2/99.8	47/53	7/93	0.2/99.8	0.352 (0.064)
rs653178-C/T	Intron, *ATXN2*	− 3.5	− 4.4	− 3.3	3.7; **5.2**; 3.3	0.05 (51.7)	3.1	2.6–3.7	0.3/99.7	47/53	7/93	0/100	0.356 (0.063)
rs847892-G/A	Intron, *ACAD10*	− 2.7	− 2.7	− 2.7	2.5; 2.5; 2.5	0.04 (49.2)	6.0	5.1–7.0	0.4/99.6	69/31	30/70	7/93	0.405 (0.080)
rs2013002-T/C	Intron, *ENST 00,000,546,840.3*	− 2.7	− 2.1	− 2.4	2.5; 1.8; 2.1	0.08 (53.2)	3.1	2.8–3.6	0.3/99.7	41/59	6/94	0.3/99.7	0.315 (0.053)

We further included in the analysis the *ALDH2* promoter variant rs886205-A/G, which is located − 360 bp from the ATG start codon of the *ALDH2* gene^51^. This promoter variant shows very large genetic differentiation with global locus-specific *F*_*ST*_ = 0.378 (s.d. = 0.055). In the 1000 Genomes data the derived allele A is the common allele in European and South Asian populations with average frequencies of about 83% and 71%, respectively. In contrast, in populations of African and East Asian ancestry the common allele is the ancestral allele G with frequencies of about 78% and 84%, respectively. For the *ALDH2* promoter variant, a study showed (in vivo and in vitro experiments) that the—360G (ancestral) allele has a significantly lower basal transcriptional activity than the − 360A (derived) allele^52^. Our LD analysis revealed that the positively selected SNPs are in complete LD (*D’* = 1) with the *ALDH2* promoter variant rs886205 (Table 2). The chromatin state data from RegulomeDB showed that the identified SNPs are associated with active transcription start site (TSS), enhancers and strong transcription in different tissues (Table 3). Importantly, the positively selected SNPs rs4766578 and rs847892 are located in the binding motif for the transcription factor *hepatocyte nuclear factor 4 alpha* (*HNF4A*). This transcription factor is an important regulatory element of *ALDH2*. The mapped phenotypes (Table 3) show that the positively selected SNPs are associated with various traits and diseases, in particular with blood pressure, cardiovascular disease, cholesterol level and autoimmune diseases. The variants rs597808 and rs2013002 are also associated with alcohol drinking and physiological traits such as blood pressure^53^. We pooled related traits (Supplementary Table 8) into four main trait category namely autoimmune diseases (AIS), blood pressure (BP), cardiovascular disease (CDS) and cancer to test the null hypothesis that the traits and the allele state are independent. We found a significant (χ^2^ = 28.828, df = 3, *p* value = 2.4e−06) relationship between the allele state and trait; the derived beneficial alleles are positively associated with AIS, BP and CDS whereas the ancestral alleles with cancer.

**Table 2:** Pairwise LD (*D*′) of SNPs under positive selection in populations of European ancestry (GBR + TSI + FIN) and the *ALDH2* promoter (*) variant rs886205; all calculated *D*′ values with *p*-value < 0.0001 (χ^2^ statistics).

Chr:pos	SNP	LD (*D′*)						
		rs3184504	rs4766578	rs10774625	rs597808	rs653178	rs847892	rs2013002
chr12:111,884,608	rs3184504	–						
chr12:111,904,371	rs4766578	1.0	–					
chr12:111,910,219	rs10774625	1.0	1.0	–				
chr12:111,973,358	rs597808	0.98	0.986	0.986	–			
chr12:112,007,756	rs653178	0.98	0.979	0.979	0.859			
chr12:112,141,570	rs847892	0.846	0.851	0.851	0.846	1.0	–	
chr12:112,200,150	rs2013002	0.956	0.970	0.97	1.0	1.0	1.0	–
chr12:112,204,427	*rs886205	1.0	1.0	1.0	1.0	1.0	1.0	1.0

**Table 3:** GTEx and RegulomeDB data on SNPs under positive selection in European populations (GBR, TSI, FIN). Given is also a summary of reported traits from the NHGRI-EBI GWAS catalogue**.** GTEx eQTLs–eGene interaction with *p* < 0.0001. RegulomeDB rank: 2b: TF binding + any motif + DNase Footprint + DNase peak; 3a: TF binding + any motif + DNase peak; 4–5: TF binding + DNase peak; 6: motif hit. The RegulomeDB probability score ranges from 0 to 1, with 1 being most likely to be a regulatory variant (for further details see^54^). Transcription factor *HNF4A*, an important regulatory element of the *ALDH2* gene expression, is given in bold.

GTEx		RegulomeDB				GWAS reported traits
eQTL	eGene	Rank	Score	Chromatin state	Motif	
rs3184504	*ALDH2, LINC01405, TMEM116*	3a	0.67022	Strong transcription; enhancers	*MTF1*	Cardiovascular disease, blood pressure, ischemic stroke, glaucoma, rheumatoid arthritis, cancer, celiac disease, type I diabetes mellitus, parental longevity, inflammatory bowel disease, multiple sclerosis, blood cell count, hypothyroidism, haemoglobin measurement
rs4766578	*ALDH2, TMEM116*	2b	0.63936	Strong transcription; enhancers	*ESRRA, ESRRB, ****HNF4A****, NR6A1*	Sjögren's syndrome, reticulocyte fraction of red cells, arthritis, vitiligo, HDL cholesterol, smoking status, coronary artery disease
rs10774625	*ALDH2, ADAM1B, TMEM116*	5	0	Strong transcription; enhancers	*FOXJ2, FOXQ1*	Hypertension, myocardial infarction, coronary artery disease, asthma, cholesterol levels, systemic lupus erythematosus, urate measurement, life span, systolic blood pressure, hypothyroidism, glomerular filtration rate
rs597808	*ALDH2, LINC01405, ADAM1B*	5	0.13454	Strong transcription	*–*	Systolic blood pressure, alcohol drinking, diastolic blood pressure, cholesterol levels, apolipoprotein B levels, colorectal cancer, allergic diseases, haematocrit, systemic lupus erythematosus, allergy
rs653178	*ALDH2, LINC01405*	4	0.60906	Active TSS; strong transcription; enhancers	*–*	Allergic disease, asthma, celiac disease, cholesterol level, eczema, Crohn's disease, chronic kidney disease, blood pressure, eosinophil counts, inflammatory bowel disease, type 1 diabetes, urate level
rs847892	*ALDH2, TMEM116, NAA25*	6	0.20016	Active TSS; strong transcription; enhancers	***HNF4A***	No data
rs2013002	*ALDH2, ADAM1B*	6	0.55195	Active TSS; enhancers	*MAFB, MAFK*	Alcohol drinking and blood pressure

## Discussion

This study provides evidence of positive selection across the human chromosomal region 12.q24.12. This finding is in line with two previous studies^55,56^. We identified seven SNPs (rs3184504, rs4766578, rs10774625, rs597808, rs653178, rs847892, rs2013002) that are under positive selection and show very large global locus-specific *F*_*ST*_ values (> 0.3), indicating high genetic differentiation between populations of European ancestry and populations from other global ancestries (Table 1). The GTEx data show that these SNPs function primarily as eQTLs for the *ALDH2* gene. We further found that this genomic region is enriched in eQTLs that influence *ALDH2* gene expression. A high number of these SNP eQTLs had significant iHS scores in the populations of European ancestry. In contrast, *cis*-eQTLs of the other genes located at chr12q24.12 showed no significant iHS values. In addition, the iHS results are supported by the iSAFE analysis which ranked the identified SNPs (eQTLs) as top-ranked mutations, with iSAFE scores > 0.304. This indicates that the target of positive selection are regulatory acting variants that influence *ALDH2* gene expression. The derived beneficial alleles at these SNP eQTLs are associated with increased expression of *ALDH2* in multiple human tissues. However, in the GTEx database, no *ALDH2 cis*-eQTLs are reported for the liver tissue. Nonetheless, the two positive selected SNPs, rs4766578 and rs847892, are located in binding sequences for transcription factor *HNF4*. That transcription factor is considered to be a master regulator of liver-specific gene expression^57^ and is an important regulatory element of *ALDH2* gene expression^35,58^. Positive selection leads to changes in the allele frequencies at the transcription factor binding sites, which could potentially lead to significant changes in the binding specificity of the liver-specific transcription factor *HNF4*. Therefore, we are inclined to hypothesize that individuals carrying the positively selected haplotypes will have higher basal expression of *ALDH2* than individuals lacking the positively selected haplotypes. In addition, the RegulomeDB data indicate that the positively selected SNPs are located in active enhancer histone marks in different tissues including the liver. Moreover, the positively selected SNP eQTLs are in complete LD with the *ALDH2* promoter variant rs847892. This promoter polymorphism influences individual differences in acetaldehyde elimination. The ancestral allele G, the common allele in populations of African and East Asian ancestry, has a lower basal transcriptional activity than the derived allele A, the common allele in populations of European and South Asian ancestry^52^. These results suggest that higher transcriptional activity and increased *ALDH2* expression in individuals of European ancestry represent a form of genetic adaptation to increased alcohol consumption, possibly enabling faster detoxification of acetaldehyde.

The derived beneficial alleles of these loci reach almost 50% in the European population, whereas in African and East Asian populations the frequencies are very low (< 0.003). The ancestral alleles at these positively selected loci appear to be fixed in ancient European hunter-gatherers, but in early farmers and individuals with steppe ancestry the frequencies of the derived alleles already range between 8 and 25%^50^. The applied Clues method found for the SNPs rs3184504, rs4766578 and rs10774625 evidence of very strong positive selection with *s* = 0.1, corresponding to an allele age of about 2.6 kya to 4.5 kya (Table 1). This is in line with the estimated timing of positive selection on the beneficial alleles in the European population GBR calculated by the Startmrca method which ranges from about 3.0 kya to 3.7 kya (except for rs847892 for which TMRCA was estimated to 6.0 kya). However, in contrast to the Startmrca method, the Clues method estimated the allele age for the other SNPs much further back in time to about 7.4 kya to 14.1 kya (again with the exception for rs847892 for which the allele age was estimated to about 21.3 kya to 30.1 kya). Nevertheless, the strong putative selection (*s* = 0.1) acting on several SNPs indicates that at these loci the alleles are much more intensely under positive selection than for example the lactase persistence locus SNP rs4988235 for which *s* = 0.0161 were calculated^59^. We further calculated the TMRCA for the East Asian-specific derived alleles rs671-A and rs3782886-C (using the Startmrca method), yielding an estimation of 5.8 kya (CI: 4.8–6.7) and 5.4 kya (CI: 4.3–6.5), respectively. Rs3782886, which is in LD with rs671, shows signals of very recent selection for the past 2000–3000 years in the Japanese population as reported in a recent study^49^. Noteworthy, rs671-A and rs1229984-A (*ADH1B* locus) were found in a subsequent study to be significantly associated with better survival in the Japanese population^60^. The estimated TMRCA for the derived alleles in our study suggests that these alleles spread in East Asia at a much earlier time than the beneficial alleles in populations of European ancestry. Archaeological evidence indicates early production of fermented alcohol in China^61^. Analysis of starch granules, phytoliths and fungi in food residues adhering to 8000–7000 year-old alcohol-making pottery vessels suggests that, in East Asia in the early Neolithic, alcoholic beverages were already being produced^62^. For Europe, archaeologically recognizable brewing material in Central European lakeside settlements show that alcoholic beverages were being produced in this region in the late Neolithic period about 6000 years ago^63^. A recent study suggests that extensive fermented alcoholic beverages such as beer were already consumed in Central Europe during the Iron Age^64^. Later, in Greek-Roman antiquity, a richly developed viticulture with high wine production was achieved and, in this period, wine became part of the daily diet of many people^65^. Alcohol consumption has apparently increased steadily since then in Europe, especially in the nineteenth century. In Germany, for example, the high level of consumption, in particular of strong spirits, in the early nineteenth century was—in analogy to the plague—referred to as Branntweinpest (brandy plague). Since the rs671-A allele leads to an inactive enzyme and thus to an excess of toxic acetaldehyde in cells with its negative physiological effects, we suggest that this allele may explain the differences in the signature of positive selection between populations of European and East Asian ancestry.

The ALDH2 enzyme plays a critical role in the detoxification of both acetaldehyde and ROS-generated aldehyde adducts such as 4-hydroxy-2-nonenal and malondialdehyde. This enzyme thus has cytoprotective effects reducing oxidative stress^66,67^. In particular, the ALDH2*2 variant (rs671-A/A), which is common only in individuals of East Asian ancestry, has been intensively studied in East Asians. While individuals with the rs671-A allele have a reduced risk of developing alcoholism, it increases their cancer risk^68,69^. Nevertheless, this allele was found to be associated in Japanese population with better survival^60^. In European populations this allele is virtually absent. In our study, however, the identified variants that are under recent positive selection in European populations act as regulatory variants and are associated with increased *ALDH2* gene expression in various human tissues*.* This suggests that individuals carrying these beneficial alleles should be more quickly able to detoxify the body from higher amounts of acetaldehyde and ROS-generated aldehyde adducts. However, a recent study reports higher methylation in alcohol-dependent patients compared to controls in the *ALDH2* promoter region^70^. Furthermore, that study suggests that positive and negative regulatory elements interact at the *ALDH2* promoter to induce genotype-mediated epigenetic changes, leading to differential transcriptional activity of this gene. In addition, a GWAS reported that the SNPs rs597808 and rs2013002, which were found in this study under positive selection, are associated with alcohol consumption and risk of developing hypertension^53^. We therefore suggest that individuals carrying the positively selected alleles may be able to consume more alcohol (over longer time periods), but may also have a higher likelihood of becoming heavy drinkers and alcohol dependent. This, then, could lead to increased methylation of the *ALDH2* promoter, resulting in decreased *ALDH2* gene expression. Accordingly, the protective effects of *ALDH2* against oxidative damage through acetaldehyde would be lost, resulting in increased risk of numerous oxidative stress-related diseases such as cancer, diabetes, inflammatory disorders and cardiovascular conditions such as hypertension and stroke. Indeed, we found that the derived beneficial alleles are positively associated with AIS, BP and CDS whereas the ancestral alleles with cancer.

To conclude, we found that very strong positive selection (with *s* ranging between 0.04 and 0.1) acts on regulatory variants affecting *ALDH2* gene expression in populations of European ancestry. Estimation of the timing of positive selection on the beneficial alleles suggests that these variants were recently adapted, approximately 3000 to 3700 years ago. The timing of selection and the signals of very strong selection make the chromosomal region chr12q24.12 one of the most intensely selected regions in the genomes of individuals of European ancestry. In contrast to the known functional consequence of the ALDH2*2 variant (rs671) in East Asians, which is associated with alcohol intolerance, in Europeans the beneficial derived alleles are associated with increased *ALDH2* gene expression. This suggests local adaptation to higher alcohol consumption in Europeans. We further hypothesize that the beneficial effects of higher *ALDH2* expression leads to an increased detoxification capacity for acetaldehyde, but possibly also to increased likelihood of chronic alcohol abuse, leading to decreased *ALDH2* expression and thus increased cell toxicity from EtOH-derived acetaldehyde as well as from ROS-generated aldehydes.

## Materials and methods

### Genomic data

We downloaded the phased genomic datasets from the 1000 Genomes project (phase 3; ftp://ftp.1000genomes.ebi.ac.uk/vol1/ftp/release/20130502/)^71^. Only 1000 Genomes data were used in this study according to the Declaration of Helsinki. We obtained SNP data from 12 human populations: three representative populations each from African ancestry (AFR), European ancestry (EUR), South Asian ancestry (SAS) and East Asian ancestry (EAS) (populations names in accordance with the 1000 Genomes project—see Supplementary Table 9). We excluded related individuals and did not include the admixed populations from the datasets because of the underlying statistical principle of the method used to detect positive selection. We used the software program PLINK 1.9^72^ (https://www.cog-genomics.org/plink/) and VCFtools v0.1.14^73^ (https://vcftools.github.io/index.html) to process the variant call format (VCF) files. We used the following filter parameters in VCFtools: *–maf 0.05* (include only sites with a Minor Allele Frequency (MAF) greater than 0.05), *–minQ 30* (include only sites with quality value above this threshold) and *–remove-indels* (exclude sites that contain an indel). Furthermore, we excluded all SNPs that deviated from Hardy–Weinberg equilibrium (with *p*-value < 1e−6) using PLINK *–hwe midp* threshold filter. We further excluded potential duplicated SNPS using bcftools version 1.10.2, (https://github.com/samtools/bcftools/) using the parameter norm *–Ov –check-ref w –fasta-ref human_g1k_v37.fasta* (ftp.1000genomes.ebi.ac.uk/vol1/ftp/technical/reference/). SNP positions are in accordance with the human genome version GRCh37/hg19 (https://genome-euro.ucsc.edu/).

### Population genetic analyses

To detect positive selection in phased genomic population data, we used the integrated Haplotype Score (iHS) approach^74^, which is implemented in the software programme selscan version 1.2.0a^75^ (https://github.com/szpiech/selscan). All scans, with default selscan model parameters, were run on phased whole chromosome data (except the Y-chromosome) with a genetic map from HapMap phase II b37^76^. The iHS approach compares extended haplotype homozygosity (EHH) values between alleles at a given SNP. It is based on the model of a selective sweep, in which a de novo adaptive mutation arises on a haplotype that is rapidly fixed in the population, thereby reducing genetic diversity around that locus^74^. The unstandardized iHS scores were normalized in default frequency bins across the entire genome using the script ‘norm’ provided by the selscan programme. Negative iHS values (iHS score < − 2.0) indicate unusually long haplotypes carrying the derived allele, and significant positive values (iHS score > 2.0) are associated with long haplotypes carrying the ancestral allele^74^. We used the Ensembl Variant Effect Predictor programme package (https://github.com/Ensembl/ensembl-vep)^77^ to map genetic information such as gene symbol and biotype to the analysed SNPs. We calculated empirical *p*-values for the obtained iHS scores across all chromosomes using R programme version 4.1.0^78^ (https://www.r-project.org/). In this study we report only results for the human chromosomal region 12q24.12, the genomic location of the *ALDH2* gene. We considered statistically significant (*p* < 0.01) iHS scores > 2.4 or < − 2.4; however, we also applied Bonferroni correction, which yields *p*-values *p* < 1 × 10^–5^ (= iHS scores > 4.2 or < − 4.2). We used the script 'colormap.plotting.R' provided by the selscan package to display the EHH plots for the SNPs that are under positive selection. Pairwise *F*_*ST*_ were calculated using Weir & Cockerham *F*_*ST*_ calculation implemented in VCFtool^73^. Negative *F*_*ST*_ values were set to zero. We calculated empirical *p*-values for the *F*_*ST*_ values (across all chromosomes) to obtain the significant threshold (*p* < 0.05) of outlier loci. In addition, locus-specific *F*_*ST*_ values and standard deviations (sd) across all analysed populations were calculated for SNPs that were detected to be under positive selection with the Genetix programme version 4.05^79^ (https://kimura.univ-montp2.fr/genetix/) applying the jackknife resampling procedure. We used the R package ggplot2^80^ to plot iHS and *F*_*ST*_ values. Allele frequency data, SNP information and ancestral/derived allele state were obtained from the Ensembl genome browser (https://www.ensembl.org/index.html)^81^. We used LDlink, a web-based application (https://analysistools.cancer.gov/LDlink/?tab=home)^82^, to explore population-specific linkage disequilibrium (LD); we report *D’* and goodness-of-fit statistics (chi-square statistics).

### iSAFE analysis

The iSAFE programme (https://github.com/alek0991/iSAFE)^56^ was used to identify beneficial mutations in the genomic region of chr12q24.12. iSAFE exploits coalescent-based signals in the surroundings of a candidate region under positive selection to rank all mutations based on their contribution to the selection signal. We used as target population under selection the European population GBR and as control populations the populations ESN (African ancestry), BEB (South Asian ancestry) and CHB (East Asian ancestry). We used the default settings for analysing a 5.6 Mbp window (9060 SNPs) surrounding the chr12q24.12 region (hg19; chr12:109,200,001–114,800,000). The developer of iSAFE showed that iSAFE scores higher than 0.304 are considered to have *p* value < 1.34 × 10^–8^. We used this cut-off as the significant threshold.

### Estimating timing of positive selection and selection coefficient (s)

We estimated the timing of selection on a beneficial allele using the R package Startmrca^29^. The method applies a Markov chain Monte Carlo simulation (MCMC) that samples over the unknown ancestral haplotype to generate a sample of the posterior distribution for the time to the most recent common ancestor (TMRCA). The model takes advantage of both the length of the ancestral haplotype on each chromosome and the accumulation of derived mutations on the ancestral haplotype to generate a sample of the posterior distribution for the TMRCA. The model requires a sample (panel) containing the haplotypes with the selected allele and a reference panel of haplotypes without the selected allele. In this study, populations of European ancestry were used as samples, and populations of the other analysed genetic ancestries were used as reference panels. Because the calculated TMRCA estimates for the populations of European ancestry were very similar regardless of the reference panels used, we report in this study only the TMRCA estimates calculated for the European populations, using the European populations both as sample panel and as reference panel. We also estimated TMRCA for the East Asian-specific functional variants rs671 (*ALDH2* locus) and rs3782886 (*BRAP* locus)^49^ using the East Asian population CHB as sample and reference panel (see Supplementary Table 9 for the corresponding population names). After normalising the TMRCA data we calculated 95% credible intervals (CI = 0.95) for the timing estimates using the equal-tailed interval method implemented in the R package bayestestR^83^. We used recombination rates from the sex-averaged recombination map from deCODE to model recombination rate variation across the human genome. We analysed 1 Mb regions up- and downstream of the genetic variants under selection with an assumed mutation rate of 1.6 × 10^–8^. We ran three independent MCMC chains, each with 25,000 iterations. We discarded the first 9000 iterations (burn–in), retaining the remaining iterations. We assumed 25 years as generation time. We further used the programme Clues (https://github.com/35ajstern/clues^59^) to estimate timing of selection and the selection coefficient (*s*) of SNPs that were identified to be under positive selection by the iHS statistics. The method relies on MCMC samples of the gene tree at the SNPs of interest. We used the programme Relate v1.1.8 (https://myersgroup.github.io/relate/^84^) to obtain this tree and to extract coalescence times. We calculated coalescence rates and effective population sizes using the integrated script *EstimatePopulationSize.sh* (obtaining the .col file); we then applied the *SampleBranchLengths.sh* module with the following parameters: *–num_samples* (number of times branch lengths sampled) 100, *–m* (mutation rate) 1.25 × 10^–8^, target region chr12q24.12 with *–first_bp* 111,700,001 *–last_bp* 112,300,000 and *–coal* (the previously obtained population size file). The output file (resample.timeb) was then used to run the Clues programme (inference.py script) with the option *–coal* (.coal file) in order to account again for population size changes and with *–tCutoff* 1000 (time to ‘cut off’ the coalescence process). We used Clues also to calculate for each SNP the selection coefficient (*s*) and the corresponding likelihood ratio (logLR) statistics^59^.

### GTEx and RegulomeDB functional data

We utilized expression quantitative trait loci (eQTLs) (accessed between May and July 2021 (dbGaP Accession phs000424.v8.p2) from GTEx Portal V8 Release (https://www.gtexportal.org/home/)^85^ to test whether any of the potential SNPs that are under positive selection function as eQTL. We included *cis*-eQTL variants within a 1 Mb window of analysed genes. The RegulomeDB database (https://regulomedb.org/)^54^ was used to obtained chromatin states; this database comprises known classes of genomic elements such as promoters, enhancers, transcription start sites, and transcription factor (TF) binding motifs. Additionally, mapped phenotype data were obtained from the NHGRI-EBI GWAS catalogue (https://www.ebi.ac.uk/gwas/)^86^ (accessed between May and July 2021).

### Consent to participate/Consent to publish

Not applicable. The 1000 Genomes data are publicly available.

### Supplementary Information

Supplementary Information 1.

Supplementary Information 2.

Supplementary Information 3.

Supplementary Information 4.

Supplementary Information 5.

Supplementary Information 6.

Supplementary Information 7.

Supplementary Information 8.

Supplementary Information 9.

## Data Availability

The gnomic data can be obtained from 1000 Genomes database. The generated iHS and Fst dataset are available from the corresponding author on reasonable request.
