# Author Correction: Positive selection acts on regulatory genetic variants in populations of European ancestry that affect ALDH2 gene expression

**DOI:** 10.1038/s41598-023-44342-w

**Published:** 2023-10-10

**Authors:** Helmut Schaschl, Tobias Göllner, David L. Morris

**Affiliations:** 1 Department of Evolutionary Anthropology, Faculty of Life Sciences, University of Vienna, Djerassiplatz 1, 1030 Vienna, Austria; 2 Department of Medical and Molecular Genetics, Faculty of Life Sciences and Medicine, King’s College London, Great Maze Pond, London, SE1 9RT UK

Correction to: *Scientific Reports* https://doi.org/10.1038/s41598-022-08588-0, published online 16 March 2022

The original version of this Article contained a repeated error, where gene name “*SH2B3*” was incorrectly given as “*SH2B2*”. As a result of this error, in the Introduction,

“This region, approximately 0.6 Mbp in size (according to the human reference genome), encompasses in addition to *ALDH2* the genes *CUX2, FAM109A, SH2B2, ATXN2, BRAP, ACAD10* and *MAPKAPK5*, as well as the uncharacterized transcript ENST00000546840.3 (UniProt F8VP50—Aldedh domain-containing protein), which partially overlaps with the genes *ACAD10* and *ALDH2*. High *F*_*ST*_ values at linked sites at the *ALDH2* locus point to some form of selection for this genomic region^36^.”

now reads:

“This region, approximately 0.6 Mbp in size (according to the human reference genome), encompasses in addition to *ALDH2* the genes *CUX2, FAM109A, SH2B3, ATXN2, BRAP, ACAD10* and *MAPKAPK5*, as well as the uncharacterized transcript ENST00000546840.3 (UniProt F8VP50—Aldedh domain-containing protein), which partially overlaps with the genes *ACAD10* and *ALDH2*. High *F*_*ST*_ values at linked sites at the *ALDH2* locus point to some form of selection for this genomic region^36^.”

And, in the Results section, under the subheading ‘Positive selection acts on regulatory variants of ALDH2’,

“We also obtained *cis*-eQTLs (1970 in total) for the other protein-coding genes located in this genomic region (*CUX2, FAM109A, SH2B2, ATXN2, BRAP, ACAD10, MAPKAPK5*) (Supplementary Table 3).”

now reads:

“We also obtained *cis*-eQTLs (1970 in total) for the other protein-coding genes located in this genomic region (*CUX2, FAM109A, SH2B3, ATXN2, BRAP, ACAD10, MAPKAPK5*) (Supplementary Table 3).”

The original Article has been corrected.

