# Supplementary material for: Positive selection acts on regulatory genetic variants in populations of European ancestry that affect ALDH2 gene expression
Source: Sci Rep. 2022 Mar 16;12:4563. doi: 10.1038/s41598-022-08588-0 (PMC8927298; doi:10.1038/s41598-022-08588-0)

**Supplementary Figure S1.** EHH plots of SNPs (chr12q24.12) under positive selection in European populations (see Table 1) that function as eQTLs for the *ALDH2* gene.


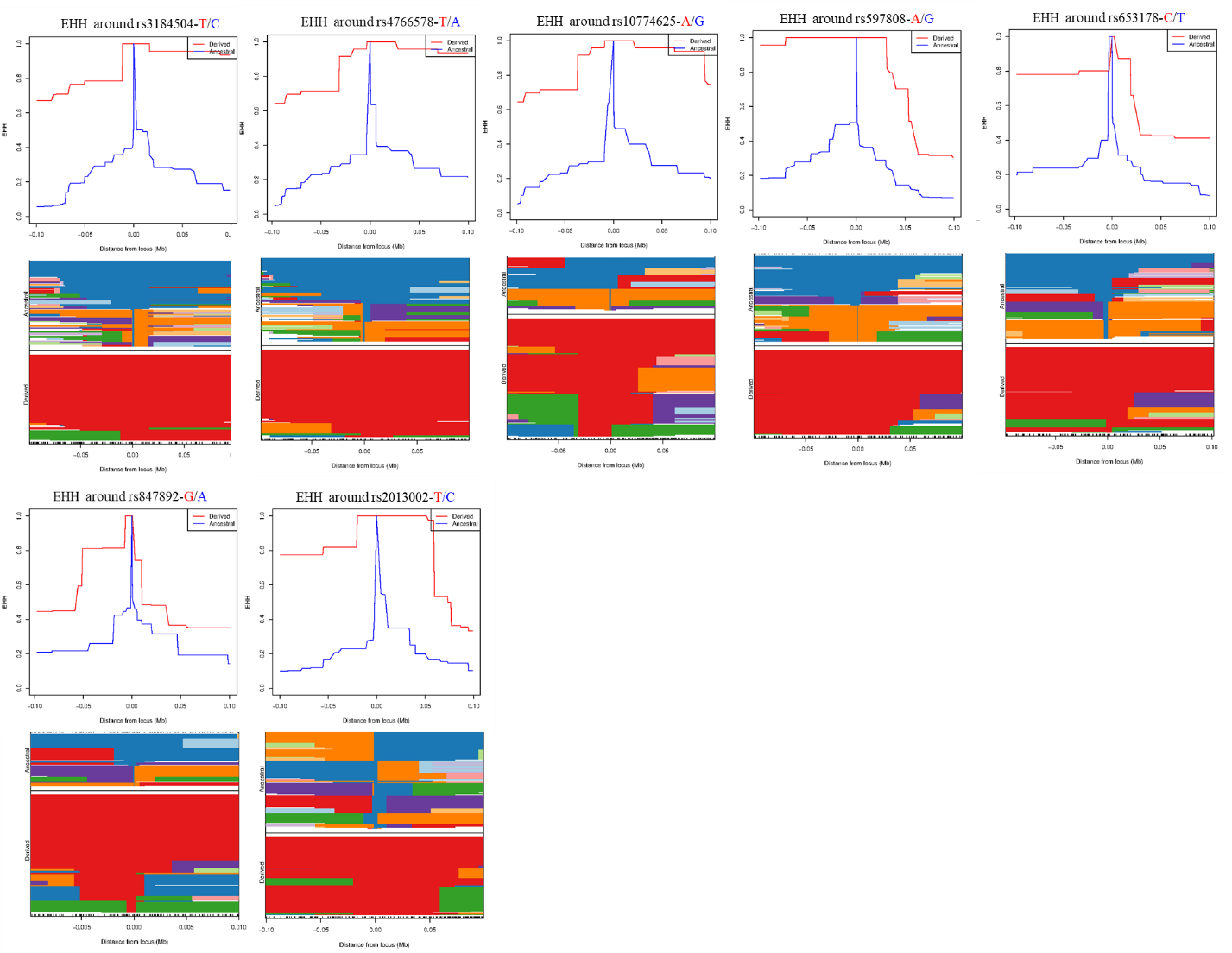

Supplement: Supplementary file 1 — Supplementary Information 1. [file 41598_2022_8588_MOESM1_ESM.docx]
