# Supplementary material for: Positive selection acts on regulatory genetic variants in populations of European ancestry that affect ALDH2 gene expression
Source: Sci Rep. 2022 Mar 16;12:4563. doi: 10.1038/s41598-022-08588-0 (PMC8927298; doi:10.1038/s41598-022-08588-0)

**Supplementary Figure S2.** Allele trajectory inferred (by the programme Clues) for the positively selected SNPs in the genomic region chr12q24.12 in population GBR (*s* = selection coefficient).


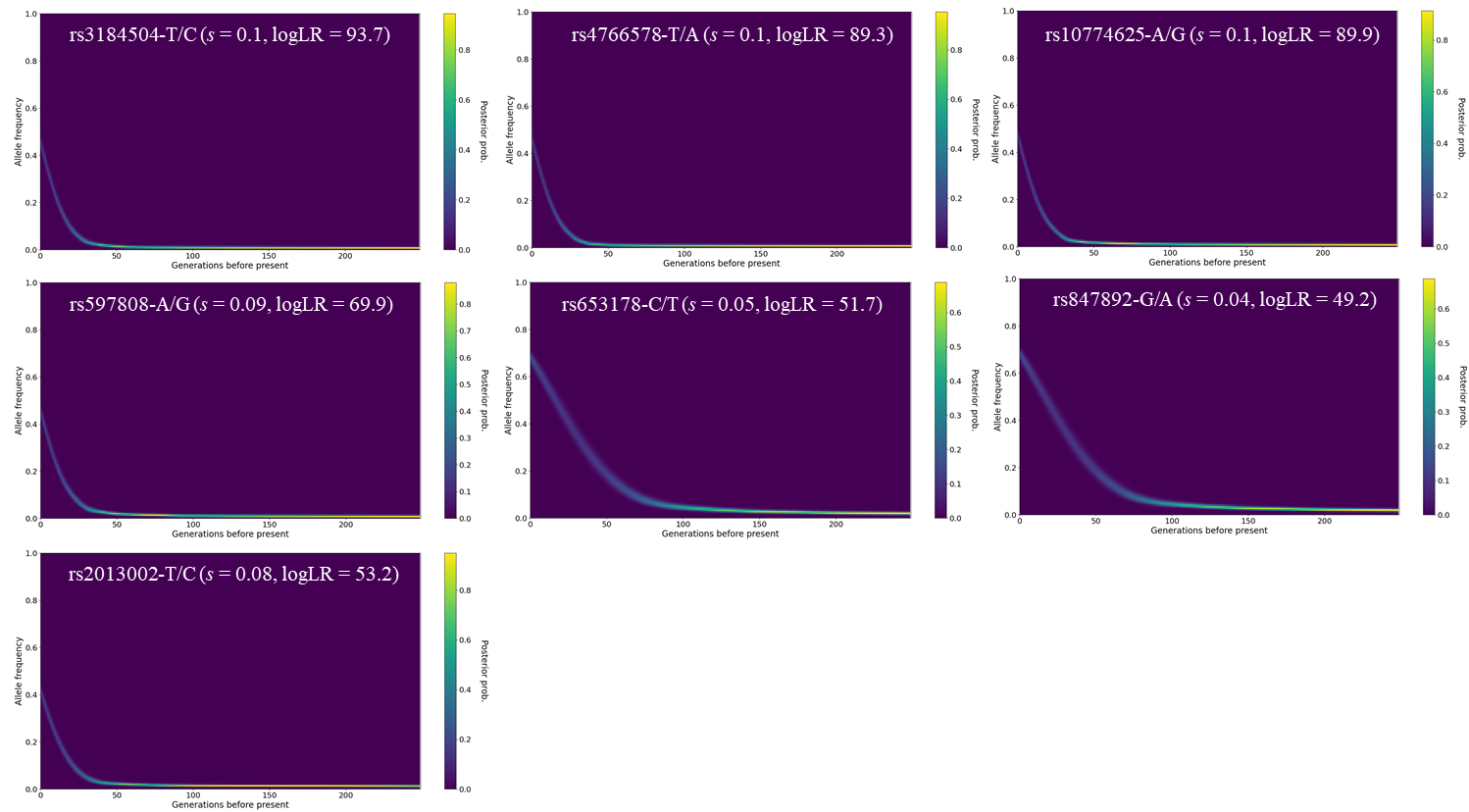

Supplement: Supplementary file 2 — Supplementary Information 2. [file 41598_2022_8588_MOESM2_ESM.docx]
