# Supplementary material for: Positive selection acts on regulatory genetic variants in populations of European ancestry that affect ALDH2 gene expression
Source: Sci Rep. 2022 Mar 16;12:4563. doi: 10.1038/s41598-022-08588-0 (PMC8927298; doi:10.1038/s41598-022-08588-0)
Supplement: Supplementary file 3 — Supplementary Information 3. [file 41598_2022_8588_MOESM3_ESM.docx]

**Supplementary Table S4.** Population pairwise *F_ST_* values of the different ancestries for SNPs that are under positive selection in European populations in the human chromosomal region 12q24.12.

|  | **Pairwise** ***F_ST_*** | | | | |
| --- | --- | --- | --- | --- | --- |
| **Positively selected SNP** | **Ancestry** | **AFR** | **EUR** | **SAS** | **EAS** |
| rs3184504 | AFR | - |  |  |  |
|  | EUR | 0.508 | - |  |  |
|  | SAS | 0.070 | 0.363 | - |  |
|  | EAS | 0.005 | 0.493 | 0.045 | - |
| rs4766578 | AFR | - |  |  |  |
|  | EUR | 0.519 | - |  |  |
|  | SAS | 0.070 | 0.375 | - |  |
|  | EAS | 0.005 | 0.504 | 0.045 | - |
| rs10774625 | AFR | - |  |  |  |
|  | EUR | 0.519 | - |  |  |
|  | SAS | 0.070 | 0.375 | - |  |
|  | EAS | 0.005 | 0.504 | 0.045 | - |
| rs597808 | AFR | - |  |  |  |
|  | EUR | 0.503 | - |  |  |
|  | SAS | 0.075 | 0.347 | - |  |
|  | EAS | 0.005 | 0.487 | 0.050 | - |
| rs847892 | AFR | - |  |  |  |
|  | EUR | 0.691 | - |  |  |
|  | SAS | 0.307 | 0.253 | - |  |
|  | EAS | 0.030 | 0.623 | 0.212 | - |
| rs4766897 | AFR | - |  |  |  |
|  | EUR | 0.403 | - |  |  |
|  | SAS | 0.026 | 0.326 | - |  |
|  | EAS | - | 0.408 | 0.0268 | - |
| rs2013002 | AFR | - |  |  |  |
|  | EUR | 0.447 | - |  |  |
|  | SAS | 0.057 | 0.318 | - |  |
|  | EAS | 0.005 | 0.431 | 0.033 | - |
