# Supplementary material for: Positive selection acts on regulatory genetic variants in populations of European ancestry that affect ALDH2 gene expression
Source: Sci Rep. 2022 Mar 16;12:4563. doi: 10.1038/s41598-022-08588-0 (PMC8927298; doi:10.1038/s41598-022-08588-0)
Supplement: Supplementary file 5 — Supplementary Information 5. [file 41598_2022_8588_MOESM5_ESM.docx]

**Supplementary Table S6**. Estimated timing (*t*) of positive selection on the derived beneficial allele in thousand years ago (kya) and 95% credible interval (CI) (rounded to one decimal figure) in different populations of European ancestry.

| **Chr** | **Beneficial allele/ ancestral allele** | **Location** | **GBR** | | **TSI** | | **FIN** | |
| --- | --- | --- | --- | --- | --- | --- | --- | --- |
|  |  |  | ***t (kya)*** | **95% CI** | ***t (kya)*** | **95% CI** | ***t (kya)*** | **95% CI** |
| **12q24.12** | rs3184504-T/C | Exon, *SH2B3* | 3.7 | 3.2–4.3 | 3.3 | 2.9–3.8 | 3.5 | 3.1–4.0 |
|  | rs4766578-T/A | Intron, *ATXN2* | 3.5 | 3.0–4.0 | 4.5 | 3.6–5.2 | 4.6 | 4.0–5.1 |
|  | rs10774625-A/G | Intron, *ATXN2* | 3.0 | 2.7–3.4 | 4.3 | 3.8–5.0 | 4.1 | 3.5–4.7 |
|  | rs597808-A/G | Intron, *ATXN2* | 3.5 | 3.0–4.1 | 4.2 | 3.7–4.8 | 3.8 | 3.2–4.3 |
|  | rs653178-C/T | Intron, *ATXN2* | 3.1 | 2.6–3.7 | 3.8 | 3.3–4.2 | 3.4 | 2.9–3.9 |
|  | rs847892-G/A | Intron, *ACAD10* | 6.0 | 5.1–7.0 | 6.1 | 5.3–7.0 | 8.3 | 6.7–9.8 |
|  | rs2013002-T/C | Intron, *ENST 00000546840.3* | 3.1 | 2.8–3.6 | 3.8 | 3.3–4.3 | 4.1 | 3.6–4.6 |
