# Supplementary material for: Positive selection acts on regulatory genetic variants in populations of European ancestry that affect ALDH2 gene expression
Source: Sci Rep. 2022 Mar 16;12:4563. doi: 10.1038/s41598-022-08588-0 (PMC8927298; doi:10.1038/s41598-022-08588-0)
Supplement: Supplementary file 6 — Supplementary Information 6. [file 41598_2022_8588_MOESM6_ESM.docx]

**Supplementary Table S7.** Estimation of timing of selection in kya (rounded to one decimal figure) calculated by Clues (for SNPs under positive selection in the European population GBR).

| **Chr** | **Beneficial allele/**  **ancestral allele** | **Location** | ***t* (kya)** | |
| --- | --- | --- | --- | --- |
|  |  |  | **age_begin** | **age_end** |
| **12q24.12** | rs3184504-T/C | Exon, *SH2B3* | 2.6 | 4.3 |
|  | rs4766578-T/A | Intron, *ATXN2* | 3.9 | 4.5 |
|  | rs10774625-A/G | Intron, *ATXN2* | 3.9 | 4.5 |
|  | rs597808-A/G | Intron, *ATXN2* | 8.9 | 12.4 |
|  | rs653178-C/T | Intron, *ATXN2* | 7.4 | 8.7 |
|  | rs847892-G/A | Intron, *ACAD10* | 21.3 | 30.1 |
|  | rs2013002-T/C | Intron, *ENST 00000546840.3* | 12.1 | 14.1 |
