# Supplementary material for: Positive selection acts on regulatory genetic variants in populations of European ancestry that affect ALDH2 gene expression
Source: Sci Rep. 2022 Mar 16;12:4563. doi: 10.1038/s41598-022-08588-0 (PMC8927298; doi:10.1038/s41598-022-08588-0)
Supplement: Supplementary file 8 — Supplementary Information 8. [file 41598_2022_8588_MOESM8_ESM.docx]

**Supplementary Table S8.** 1000 Genomes populations and genetic ancestries included in the study (<ftp://ftp-trace.ncbi.nih.gov/1000genomes/ftp/release/20130502/>).

| **Number of individuals** | **Population code** | **Population**  **description** | **Super population** | **Super population code** |
| --- | --- | --- | --- | --- |
| 99 | LWK | Luhya in Webuye, Kenya | Africa | AFR |
| 113 | GWD | Gambians from The Gambia | Africa | AFR |
| 99 | ESN | Esan in Nigeria | Africa | AFR |
| 91 | GBR | British in England and Scotland | Europe | EUR |
| 99 | FIN | Finnish in Finland | Europe | EUR |
| 107 | TSI | Toscani in Italia | Europe | EUR |
| 86 | BEB | Bengali from Bangladesh | South Asian | SAS |
| 102 | ITU | Indian Telugu from the UK | South Asian | SAS |
| 96 | PJL | Punjabi from Lahore, Pakistan | South Asian | SAS |
| 103 | CHB | Han Chinese in Beijing, China | East Asian | EAS |
| 104 | JPT | Japanese in Tokyo, Japan | East Asian | EAS |
| 99 | KHV | Kinhin Ho Chi Minh City, Vietnam | East Asian | EAS |
